# Targeting bone morphogenetic protein signalling in midbrain dopaminergic neurons as a therapeutic approach in Parkinson's disease

**DOI:** 10.1042/NS20170027

**Published:** 2017-03-31

**Authors:** Gerard W. O'Keeffe, Shane V. Hegarty, Aideen M. Sullivan

**Affiliations:** 1 Department of Anatomy & Neuroscience, University College Cork, Cork, Ireland; 2 APC Microbiome Institute, University College Cork, Cork, Ireland

**Keywords:** axon, bone morphogenetic protein, dopamine, neuron, neuroprotection, neurotrophic factor, Parkinson's disease, Smad

## Abstract

Parkinson's disease (PD) is the second most common neurodegenerative disease, characterized by the degeneration of midbrain dopaminergic (mDA) neurons and their axons, and aggregation of α-synuclein, which leads to motor and late-stage cognitive impairments. As the motor symptoms of PD are caused by the degeneration of a specific population of mDA neurons, PD lends itself to neurotrophic factor therapy. The goal of this therapy is to apply a neurotrophic factor that can slow down, halt or even reverse the progressive degeneration of mDA neurons. While the best known neurotrophic factors are members of the glial cell line-derived neurotrophic factor (GDNF) family, their lack of clinical efficacy to date means that it is important to continue to study other neurotrophic factors. Bone morphogenetic proteins (BMPs) are naturally secreted proteins that play critical roles during nervous system development and in the adult brain. In this review, we provide an overview of the BMP ligands, BMP receptors (BMPRs) and their intracellular signalling effectors, the Smad proteins. We review the available evidence that BMP–Smad signalling pathways play an endogenous role in mDA neuronal survival *in vivo*, before outlining how exogenous application of BMPs exerts potent effects on mDA neuron survival and axon growth *in vitro* and *in vivo*. We discuss the molecular mechanisms that mediate these effects, before highlighting the potential of targeting the downstream effectors of BMP–Smad signalling as a novel neuroprotective approach to slow or stop the degeneration of mDA neurons in PD.

## Introduction

Dopamine is one of the major catecholaminergic neurotransmitters found in the adult brain. Seventy-five percent of all dopaminergic neurons are found in the midbrain, with an estimated 400000 to 600000 in the human midbrain [1–3]. During development, the generation and differentiation of dopaminergic neurons are regulated by a complex, temporally regulated pattern of gene expression that ultimately assembles three major dopaminergic circuits that are crucial for normal behaviour and physiological function [4,5]. These circuits are formed by the arrangement of midbrain dopaminergic (mDA) neurons into three different populations known as the A8, A9 and A10 groups [6]. mDA neurons in the A10 and A8 clusters are, respectively, found in regions known as ventral tegmental area (VTA) and the retrorubral field (RRF). VTA and RRF mDA neurons project to the nucleus accumbens, limbic system and prefrontal cortex via the mesocortical and mesolimbic projections to regulate emotion, motivation and reward behaviours [7]. Consequently, alterations in mesocortical and mesolimbic mDA neuronal functioning have been implicated in a range of psychiatric disorders including schizophrenia, drug addiction and depression [8,9]. The other population of mDA neurons, the A9 population, is located in a region of the midbrain called the substantia nigra pars compacta (SNpc). These A9 mDA neurons project to and innervate the dorsal striatum (caudate-putamen) to form the nigrostriatal pathway, which has a well-established role in regulating motor function. It should be noted however that the A9 mDA neuronal population may also play significant roles in reward function [10] (for detailed review see [11]). A9 mDA neurons have been the focus of a considerable research effort, as the progressive loss of these neurons is one of the core pathological features of the neurodegenerative disorder, Parkinson's disease (PD) [12–15]. Despite much progress in understanding the pathological basis of PD and the molecular mechanisms involved [16], there is still no disease-modifying therapy.

Though various experiment therapies have been proposed for PD, the application of neurotrophic factors to the PD brain has been the focus of intensive investigation [17]. Several members of the transforming growth factor-β (TGFB) superfamily are potent neurotrophic factors for mDA neurons [18]. These include glial cell line-derived neurotrophic factor (GDNF) and neurturin (NTN), which have been tested in clinical trials [19]. Despite initial promising results, these trials have not to date been successful [20,21]. Recent work has shown that the lack of efficacy of GDNF and NTN may result from alpha-synuclein-induced down-regulation of their common co-receptor, Ret, which is crucial for GDNF and NTN signalling [22] as GDNF did not confer neuroprotection in the α-synuclein rat model of PD [23]. As such the authors of the latest adeno-associated virus (AAV)–NTN trial stated that “better results might be achieved with other trophic factors that are not RET dependent” [21]. BMPs have been shown to exert neurotrophic effects on mDA neurons in a Ret-independent manner.

In this review, we will: provide an overview of the bone morphogenetic proteins (BMPs) and their intracellular signalling effectors, the Smads; review the evidence that these morphogens play an endogenous role in mDA neurons; review the effects of BMPs on mDA neuron survival and axon growth; and highlight the potential of application of BMP ligands or molecular targeting of Ret-independent BMP–Smad signalling as a potential neuroprotective approach to attenuate or stop the degeneration of A9 mDA neurons in PD.

## BMP–Smad signalling

The TGFB superfamily is a large family of related growth factors comprised of at least 30 members in mammals. This superfamily is subdivided into two functional groupings known as the TGFB-like group that includes TGFBs, activins, nodals and some growth and differentiation factors (GDFs), and the BMP-like group that includes the BMPs, most GDFs and anti-Mullerian hormone (AMH) (for excellent review see [24]). The BMPs and GDFs constitute the largest subgroup of the TGFB superfamily, and BMP–Smad signalling functions in many crucial aspects of neural development [25]. Although members of both the TGFB-like and BMP-like groups have been examined as neurotrophic factors for mDA neurons [18,26], for the purposes of this review we will focus on three members of the BMP-like group that have been extensively studied in this regard, namely BMP2 (HGNC:1069), BMP7 (HGNC:1074) and GDF5, which is also known as BMP14 (HGNC:4220).

**Figure 1:**
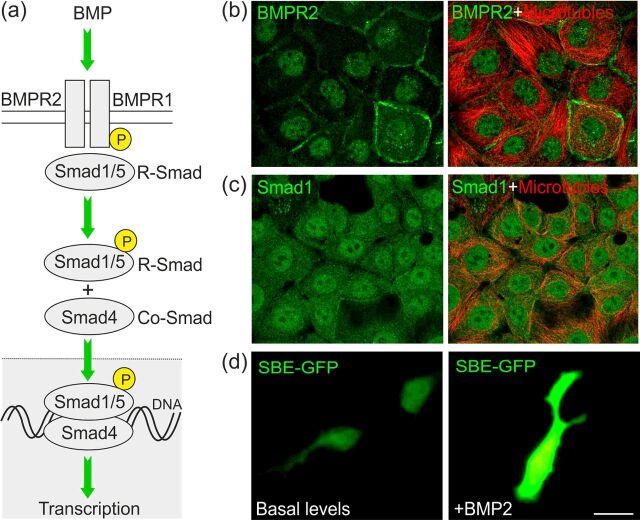
The canonical BMP–Smad signalling pathway (**a**) Binding of BMP dimers induces heteromeric complex formation and activation of type I and type II BMPR via transphosphorylation. These recruit R-Smads (Smad1/5) that, when phosphorylated, form heteromeric complexes with Co-Smad4, then translocate to the nucleus, bind DNA and alter transcription. (**b** and **c**) Immunocytochemistry showing the subcellular localization of BMPR2 (green) and Smad1 (green) respectively, in A-431 and MCF7 cells stained for microtubules (red). Image credit: Human protein atlas www.proteinatlas.org [87]. Image data available from v16.proteinatlas.org at the following URLs: www.proteinatlas.org/ENSG00000204217-BMPR2/cell#human and www.proteinatlas.org/ENSG00000170365-SMAD1/cell#human. (**d**) Representative images of SH-SY5Y cells transfected with a Smad-binding element (SBE)–GFP reporter plasmid, showing that BMP treatment leads to Smad-dependent transcription [34,38]; scale bar=10 μm.

BMP ligands, as all TGFB superfamily members, signal through a canonical pathway involving a heteromeric complex of type I (ACVR1, BMPR1A and BMPR1B) and type II (ACVR2A, ACVR2B and BMPR2) transmembrane serine/threonine kinase receptors that phosphorylate and activate intracellular effector proteins called Smads (Figure 1a) [24]. These receptors can pair in various combinations with varying affinities for BMP ligands, but BMP2, BMP7 and GDF5 can bind BMPR2 and ACVR2A and 2B [27,28], while BMP2 and BMP7 bind BMPR1A or BMPR1B receptors with high affinity [29]. Interestingly, GDF5 binds BMPR1B and BMPR2, but not BMPR1A, with high affinity (Figure 1b) [30]. BMP ligand binding results in type-I receptor phosphorylation that recruits and phosphorylates receptor-activated Smad proteins (R-Smads) (Figure 1c). These BMP-activated R-Smads (Smad1, Smad5 and Smad8/9) complex with Smad4, after which this transcriptional complex accumulates in the nucleus, binds to DNA and elicits a transcriptional response (Figure 1d). It should be noted that this is an overly simplistic view, as BMP signalling is complex and is regulated at multiple levels through a number of different mechanisms (for detailed reviews see [24,25]). In recent years, it has also emerged that BMPs can have context-dependent effects through non-canonical pathways that include GTPases, MAPK and PI3K pathways [31–33]). Since the effects of BMPs on mDA neurons are largely Smad-dependent [34,35], for the purposes of this review we will focus on describing the contribution of the canonical BMP–Smad pathway to mDA neuronal survival and growth; however, understanding the role of non-canonical BMP signalling is an important line of future investigation.

## A role for endogenous BMP signalling in mDA neurons

The physiological role of BMP signalling in mDA neurons remains unclear, yet a number of lines of evidence suggest that it is actively involved. Indeed, we have found that *Bmpr2* mRNA is stably expressed at high levels in rat midbrain from embryonic day (E)14 through to adulthood [34]. Conversely, *Bmpr1b* mRNA is expressed at low levels at E14 rat midbrain, but there is a significant increase in its expression with advancing age that reaches maximal levels in adulthood [34]. BMPR2 and BMPR1b are co-expressed in mDA neurons, as both receptors co-localize with tyrosine hydroxylase (TH)-stained embryonic and adult rat mDA neurons [34]. These data suggest that endogenous BMP signalling may play a functional role in mDA neurons *in vivo*.

The strongest line of evidence supporting such a role comes from a study of BMPR2 dominant negative (BMPR2DN) mice [36]. Adult male BMPR2DN mice had a 20% reduction in mDA neuron number, but an almost complete loss (approximately 90%) of striatal innervation. BMPR2DN mice also demonstrated reduced locomotor activity compared with their wild-type counterparts [36]. However as BMPR2 is strongly expressed throughout life [34], it is unclear whether this reflects a role for BMP–Smad signalling in the development of mDA neurons, or a role in their maintenance. While the two possibilities are not mutually exclusive, this is an important question to resolve, as BMPs also play roles in the early development of other TH-positive (TH^+^) adrenergic neurons, which are also affected in PD. Specifically, inhibition of global BMP signalling in the chick embryo by the BMP antagonist Noggin prevented sympathetic neuron generation [37]. Moreover, there is regional failure of sympathetic innervation, but not survival, in mice that are either heterozygous or homozygous for the *Gdf5* ^bp^ null mutation [38], a frame-shift mutation in the *Gdf5* gene [39]. Specifically, there were sympathetic innervation defects without a change in the number of the innervating neurons. This suggests that the nigrostriatal deficiencies seen in BMPR2DN mice may reflect a role for BMP–Smad signalling in mDA neuronal development. With the generation of a number of *BMPR^flox/flox^* and *Smad^flox/flox^* mouse lines (for review see [25]), injection of AAV encoding for Cre (AAV-Cre) to the SNpc of these mice would allow the contribution of BMP–Smad signalling to the maintenance of mDA neurons during adulthood to be determined. The feasibility of this approach is highlighted in a recent elegant study that used intrastriatal injection of AAV-Cre in *GDNF^flox/flox^* mice to examine the contribution of target-derived GDNF to mDA neuron maintenance [40]. There has been only one study that examined the BMP ligands, which showed that adult *Bmp7^+/−^* heterozygous mice (*Bmp7^−/−^* mice die with 24 h of birth) had a subtle loss of nigrostriatal innervation and were more vulnerable to methamphetamine-induced mDA neuronal injury [41]. This suggests that endogenous BMP signalling has a significant role in protecting and maintaining adult mDA neurons *in vivo.* However, further study is required to delineate the exact role of BMP–Smad signalling in mDA neuron development and/or maintenance, and to understand the relative contribution and functional redundancy between BMP ligands and receptors in these processes.

## Somal and axonal degeneration in PD

The progressive loss of A9 mDA neurons in PD leads to a loss of dopaminergic innervation and consequently dopamine levels in the striatum. While there has been a historical focus on protecting the cell bodies of mDA neurons, there is a growing awareness that early axonal degeneration may be central to PD progression (for review see [42,43]). A quantitative study in humans has shown that there was a modest loss of TH^+^ dopaminergic axons in the post-commissural putamen (striatum) at 1 year after PD diagnosis, while by 4 years post diagnosis there was a complete loss of dopaminergic innervation [12]. Interestingly, while there was a marked reduction in mDA neurons in the SNpc (50–90%) at the earliest times post diagnosis, there was little subsequent loss, and a residual population of mDA neurons was still observed decades after diagnosis [12]. Therefore, it appears that while the loss of dopaminergic innervation occurs rapidly and is completed by 4 years post diagnosis, the degeneration of mDA neurons lags behind the axonal loss. When considering potential therapies for PD, it thus may not be sufficient to stop the degeneration of the neuronal soma. Protecting the axons of the residual mDA neuronal population may also be an important approach, given that a single A9 mDA neuron has a widespread and highly dense axonal arborization that can cover approximately 2% of the striatum [44]. Moreover, the molecular mechanisms of degeneration of the neuronal soma may be distinct from those that lead to axonal degeneration, as highlighted in animal models of PD.

Stereotaxic injection of 6-hydroxydopamine (6-OHDA) into the medial forebrain bundle (MFB), striatum or SN of adult rats induces the selective death of nigrostriatal dopaminergic neurons and is a commonly used animal model of PD. Intrastriatal injection of 6-OHDA normally results in a rapid degeneration of mDA neurons in the SNpc and their axonal terminals in the striatum, depending on the time the tissue is analysed after introduction of the toxin [45]. Interestingly, there is complete protection of mDA neurons following intrastriatal 6-OHDA in *Jnk2^−/−^* and *Jnk3^−/−^* mice but no protection of their axonal terminals [46], suggesting that there are distinct mechanisms that mediate the degeneration of mDA neuron soma and of their axons [42,47,48]. This highlights the need to better identify the molecular mechanisms that regulate mDA neuron survival and axon growth, and to translate this into new neuroprotective therapies. Here, we review the evidence showing that BMPs can protect mDA neurons by promoting both mDA neuron survival and axon growth *in vitro* and *in vivo.*

## BMPs as neurotrophic factors for mDA neurons

The initial evidence for BMPs acting as neurotrophic factors for mDA neurons came from a study showing that treatment of embryonic day E14 rat ventral mesencephalon (VM) cultures with BMP ligands led to significant increase in mDA neuron survival. Specifically, treatment of these cultures with 10 ng/ml of BMP2 or BMP7 led to a 1.5-fold increase in the number of mDA neurons after 7–8 days [49,50]. Interestingly, both BMP2 and BMP7 also increased the number of astrocytes in these cultures, and inhibition of this increase in astrocyte number using the anti-mitotic agents 5-fluorodeoxyuridine and α-aminoadipic acid inhibited the survival-promoting effects of BMP7 [49]. Similarly, GDF5 also increased mDA neuronal survival and astroglial proliferation in E14 rat VM cultures [51–53]. However, inhibition of astroglial proliferation with antimitotic agents in GDF5- [54] or BMP2- [50] treated VM cultures did not eliminate the survival-promoting effects of these BMPs. BMP2 and GDF5 also increased neuronal complexity and promoted increases in neurite length and branching of mDA neurons in these cultures [50,52,55]. These data suggested a direct effect of BMPs on mDA neurons, but the underlying molecular mechanisms and the contribution of canonical Smad signalling to these effects remained unknown.

**Figure 2:**
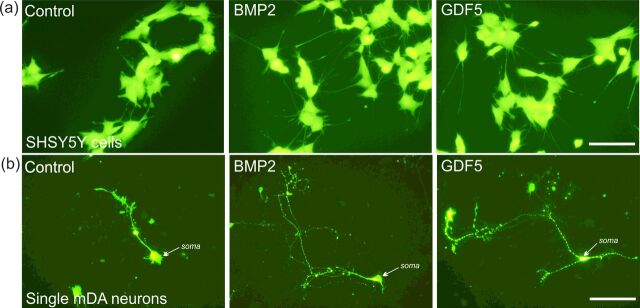
BMPs are potent inducers of neurite growth in *in vitro* cellular models (**a**) Representative photomicrographs of neurite length in BMP2- and GDF5-treated SH-SY5Y cells stained with the vital fluorescent dye Calcein after 4DIV [35]. (**b**) Representative photomicrographs of single TH^+^ mDA neurons prepared from E14 rat VM, treated with 10 ng/ml BMP2 or GDF5, as indicated, for 4DIV [34]; scale bar=100 μm.

To begin to address this, we studied the effects of BMP2 and GDF5 (as the neurotrophic effects of these ligands were not glial-dependent) using SH-SY5Y cells, a widely-used *in vitro* cell model of human dopaminergic neurons [56,57]. This cell line expresses for BMPRs, Smad-1/5 and co-Smad4, and treatment of SH-SY5Y cells with BMP2 or GDF5 led to a significant increase in total neurite length (Figure 2a) [35,58]. Moreover, there was a significant increase in phosphorylated (p)-Smad1/5 levels within 1 h of treatment [35]. Interestingly, the effects of BMP2 and GDF5 on p-Smad1/5 and neurite growth were prevented by co-treatment with dorsomorphin, a small molecule inhibitor of BMPR1A/B [35,59]. This suggested that the effects of BMP2 and GDF5 on neurite growth were mediated through canonical BMPR-Smad signalling. In agreement with this, transfection of SH-SY5Y cells with either a pcDNA plasmid expressing a constitutively active BMPR1B (caBMPR1B) [60] or a control plasmid demonstrated that caBMPR1B induced significant increases in p-Smad1/5 and neurite growth [35]. These effects were blocked by co-transfection with siRNAs targeting the co-Smad4, to inhibit nuclear translocation of the Smad transcriptional complex [35]. These data showed that BMPR1B-mediated activation of Smad signalling promoted neurite growth in SH-SY5Y cells.

However, it should be noted that while SH-SY5Y cells are useful models for studying molecular mechanisms, they are ultimately neuroblastoma cells that recapitulate some, but not all, features of mDA neurons. To address this directly in mDA neurons, we studied individual mDA neurons in primary cultures of E14 rat VM. Similar to SH-SY5Y cells, treatment with either BMP2 or GDF5 increased p-Smad1/5 levels and promoted survival [53] and neurite growth in TH^+^ mDA neurons (Figure 2b) [34]. These effects of BMP2 and GDF5 were blocked by co-treatment with Noggin and dorsomorphin, and mimicked by transfection with caBMPR1B. Moreover, transfection of individual cells with siRNAs targeting BMPR1B or Smad4 prevented these effects [34]. These data showed that BMP ligand binding to BMPR activates Smad signalling, which exerts a direct effect to promote axon growth in mDA neurons. Given these findings and the significant loss of mDA axons in PD, targeting BMP–Smad signalling may be a useful therapeutic approach to slow or prevent the loss of axons, or to promote the regrowth of axons from the residual population of mDA neurons [12] that remain in the midbrain throughout the disease duration.

Despite these findings, it remains unclear whether the survival-promoting effects of BMPs on mDA neurons are dependent on canonical Smad signalling [49–52]. This is an important question for future research given that there may be distinct molecular mechanisms that regulate neuronal survival and axonal maintenance in mDA neurons [46]. For example, a recent study that used another population of adrenergic neurons has shown that while GDF5, acting in a retrograde manner, promoted significant increases in axonal growth and branching of sympathetic neurons (which are also affected in PD), it had no effect on their survival [38]. These effects were also mimicked by the transfection of individual neurons with caBMPR1b. Moreover, the O'Keeffe et al. also directly tested the requirement of Smad-dependent gene transcription for neurite growth, by co-transfecting with caBMPR1B and either a decoy dsDNA-oligonucleotide containing the Smad consensus binding sequence (5′-gtacattgtcagtctagacataact-3′), which sequesters activated R-Smads and prevents them from binding to DNA (Smad decoy) or a control oligonucleotide with a scrambled sequence (control decoy). Similar to the effects in SHSY5Y cells [35] and mDA neurons [34], the transfection of individual sympathetic neurons with caBMPR1B led to highly significant increase in neurite length, branching and overall neurite arbour size and complexity, effects that were completely prevented by the Smad decoy but not by the control decoy. This shows that the activation of canonical Smad signalling promotes axon growth directly in neurons that are affected in PD. However, the contribution of canonical BMP–Smad signalling to mDA neuron survival is an important question for future research. While the axon growth-promoting effects of BMPs may be dependent on their canonical Smad signalling pathway, whether this is the case for mDA neuron survival currently remains unknown.

## Neuroprotective effects of BMPs in animal models of PD

In order to examine the potential of neurotrophic factors for clinical use in PD, studies on animal models are crucial. Both GDF5 and BMP7 have been shown to induce neuroprotective effects on the nigrostriatal dopaminergic pathway and to confer improvements in motor function in 6-OHDA rodent models of PD (Table 1).

**Table 1:** Summary of the *in vivo* studies using BMPs in animal models of PD Rotational (circling) behaviour in response to i.p. injection of amphetamine is widely used to assess the effects of treatments in rat models of PD. Rats with a unilateral lesion of the nigrostriatal pathway display a distinct bias for the contralateral limbs, resulting in profound rotational behaviour in a direction ipsilateral to the lesion following administration of amphetamine, which induces synaptic dopamine release, thus stimulating locomotion. The symbol ‘↓ Rotations’ refers to a decrease in the mean number of amphetamine-induced rotations displayed by the rats, indicating a protective effect on the lesioned nigrostriatal pathway.

Author, date (reference number)	Neurotoxin (location)	Neurotrophic factor	Substantia nigra	Striatum	Motor function
Sullivan, 1997	6-OHDA (MFB)	GDF5 (SN + LV)	↑mDA neuron survival	↑DA levels ↑DA turnover ↑DA terminals	↓Rotations
Sullivan, 1999	6-OHDA (MFB)	GDF5 (SN or striatum or LV)	↑mDA neuron survival	↑DA levels ↑DA turnover ↑DA terminals	↓Rotations
Sullivan, 1998	6-OHDA (MFB)	GDF5-treated E14 VM grafts (striatum)	–	–	↓Rotations
Hurley, 2004	6-OHDA (striatum)	GDF5 (SN or striatum)	↑mDA neuron survival	No effect on DA terminals	↓Rotations
O'Sullivan, 2010	6-OHDA (striatum)	GDF5-overexpressing E13 VM cells (striatum)	–	–	↓Rotations
Costello, 2012	6-OHDA (MFB or striatum)	GDF5-overexpressing CHO cells (SN or striatum)	↑mDA neuron survival	–	↓Rotations
Zuch, 2004	6-OHDA (striatum)	BMP7 (LV)	↑TH immunoreactivity	↑DA levels	No effect on locomotor activity
Harvey, 2004	6-OHDA (MFB)	BMP7 (SN)	↑TH immunoreactivity	–	↓Rotations
Espejo, 1999	6-OHDA (SN)	BMP2-treated E14 VM grafts (striatum)	–	–	↓Rotations

Since BMPs do not cross the blood–brain barrier (BBB), it is necessary to administer these proteins directly into the brain to examine their effects *in vivo*. Several studies have reported the potent neuroprotective effects of stereotaxic injection of recombinant human (rh)GDF5 into the 6-OHDA-lesioned adult rat brain. Intracerebral injection of rhGDF5 protein has been shown to protect nigral dopaminergic neurons and their striatal terminals from 6-OHDA lesion of the MFB [61,62]. In the clinical situation, PD patients have already experienced significant degeneration of dopamine neurons prior to receive treatment. Thus, potential therapies are required to achieve restoration of the nigrostriatal pathway in animal models. Injection of rhGDF5 protein into the striatum at one or two weeks after intrastriatal 6-OHDA lesion has been shown to restore nigrostriatal dopaminergic integrity and function [63]. BMP proteins are rapidly bio-metabolized in the brain and thus in order to explore the therapeutic potential of these agents, it is important to investigate methods of achieving long-term delivery. Studies that implanted GDF5-overexpressing embryonic mDA neurons [53] or an rhGDF5-overexpressing cell line [64] into the adult rat striatum showed significantly preservation of the nigrostriatal mDA neuronal pathway after 6-OHDA lesions, with corresponding enhancement of motor function.

BMP7 has also been reported to have neuroprotective effects in *in vivo* PD models. In the intrastriatal 6-OHDA lesion rat model, intraventricular injection of BMP7 at one week post-lesion protected nigral dopaminergic neurons, but had no effect on locomotor activity [65]. Another study found that pre-treatment with intranigral BMP7 protected nigral dopaminergic neurons and striatal dopamine release from 6-OHDA-induced lesions of the nigrostriatal pathway, as well as improving motor function in these rats [66]. These discrepancies may be as a result in differences in the timing and site of BMP7 application among these studies.

One therapeutic approach for PD is cell replacement using grafting of embryonic mDA neurons into the adult brain to replace those neurons that have been damaged by the disease. A limitation of this type of therapy is the low survival rate of the grafted cells. A large research effort has investigated agents that could be used to improve cell survival and several neurotrophic factors have shown promise in this application. Pre-treatment with either GDF5 [53,67] or BMP2 [68] has been shown to confer survival-promoting and function-enhancing effects on embryonic rat mDA neurons after transplantation into the 6-OHDA-lesioned adult rat striatum.

The above studies illustrate the potential of the BMPs in the treatment of PD. However, the use of proteins as therapeutic agents is limited by their rapid metabolism in the brain. Furthermore, neuroprotective agents are generally more efficacious at earlier stages of the disease, when the nigrostriatal pathway is relatively intact. Therefore the potential effectiveness of BMP therapies declines as the disease progresses, and thus methods that ensure a sustained and targeted supply of these agents to nigrostriatal dopaminergic neurons must be developed. One such approach under investigation is gene therapy, which aims to introduce a long-term source of neurotrophic proteins to the brain, and which has shown promise for PD (for review see [19]). Gene therapy using viral vectors to achieve an adequate supply of BMPs to degenerating nigrostriatal neurons in the PD brain may be an optimal way of ensuring clinical efficacy, even in advanced disease states. The neurotrophic factors GDNF and NTN have shown some promise in clinical trials in PD patients, although problems with delivery, targeting and patient selection have meant that their full potential has yet to be achieved (for review see [69]).

## Small molecule approaches for targeting BMP–Smad signalling

At present, direct injection into the brain is the only practical method of administrating viral vectors carrying neurotrophic factors. While this approach is safe and clinically feasible in patients [70,71], it is reliant upon mDA neurons in the PD brain maintaining expression of the full complement of proteins required to respond to the neurotrophic factor. This challenge was highlighted by comprehensive studies that showed that GDNF failed to exert neurotrophic effects on mDA neurons in the rat α-synuclein model of PD [23]. GDNF could not exert its neurotrophic effect in this model due to α-synuclein-induced down-regulation of the GDNF receptor, Ret [55]. Furthermore, qualitative studies in humans suggest that Ret is down-regulated in PD [22]. One approach to circumvent this problem would be to bypass the need for neurotrophic factors or their receptors by selectively activating their downstream effectors. Thus, identification of the signalling cascades that mediate the effects of neurotrophic factors may allow the development of ligand–receptor-independent, non-invasive molecular therapies using small molecules or biologics that cross the BBB. While this approach can be applied to any neurotrophic factor, we have recently begun to examine the neuroprotective potential of targeting the downstream effectors of the BMP ligands in mDA neurons. Although several inhibitors of canonical BMP-signalling pathway are known, including the synthetic antagonist dorsomorphin [59], to date very few small molecules have been shown to activate canonical BMP signalling.

p300/CBPs are histone acetyltransferases (HATs) that act as transcriptional co-activator proteins, playing key roles in the regulation of levels of transcriptional activity in response to diverse physiological stimuli [72]. p300/CBPs have been shown to interact with Smad1 and Smad4 [73], and to activate BMP–Smad-mediated gene transcription [74]. As Smad-mediated gene transcription is crucial for at least the axon growth-promoting effects of BMPs [38], targeting p300/CBPs to activate BMP–Smad-mediated gene transcription may exert BMP-like neurotrophic effects in mDA neurons. To provide proof-of-principle for this approach, we recently used a selective and potent small molecular activator of p300/CBPs, known as CTPB (*N*-(4-chloro-3-trifluoromethyl-phenyl)-2-ethoxy-6-pentadecyl-benzamide) [75]. Treatment of SH-SY5Y cells with 5 μM CTBP led to a 1.5-fold increase in neurite growth [76], which is the same level as that induced by GDF5 and BMP2 in SY-SY5Y cells [35] and mDA neurons [34]. Moreover, CTPB protected against 6-OHDA-induced cell death in SY-SY5Y cells [76]. Although p300/CBP interacts with Smad1/Smad4 [73], which are required for the axon growth-promoting effects of GDF5 [34,35,38], and activating p300/CBP activates BMP–Smad-mediated gene transcription [74], it will be important to determine if the neurotrophic effects of CTBP are dependent upon Smad-mediated gene transcription. Despite this, CTPB is a promising molecule for targeted induction of p300/CBP activity *in vivo*, and ongoing studies will explore strategies for its delivery to the brain. For example, CTPB conjugated to carbon nanospheres readily crosses the BBB and can localize in the brain [77]. Future studies will focus on determining the neuroprotective efficacy of this approach in animal models of PD.

Another molecular approach to activate BMP–Smad-dependent gene transcription in neurons may be to use histone deacetylase (HDACs) inhibitors (HDIs). HDIs are potent transcriptional modulators that inhibit HDACs to increase transcription and cross the BBB [78]. The neurotrophic effects of HDIs on mDA neurons have been well documented [79–83], and we have recently shown that a class-IIa HDI promotes axon growth in SH-SY5Y cells, mDA and sympathetic neurons, and protects them against MPP^+^-induced degeneration [84]. While it is likely that diverse molecular mechanisms may mediate these effects, class-IIa HDAC inhibition led to a significant increase in the phosphorylation of Smad-1/5 in all three cell types [84]. This suggests that HDIs, which can cross the BBB, can increase basal Smad-signalling in neurons in the absence of an exogenous BMP ligand. In future work, it will be important to determine the contribution of increase in Smad signalling to the neurotrophic effects of HDIs on mDA neurons. There is significant potential in a small molecule approach for clinical translation in neurodegenerative diseases. This is highlighted by a Phase I trial investigating the ability of glycerol phenylbutyrate (an FDA-approved HDI) to increase the removal of α-synuclein from the brain (NCT02046434) (https://clinicaltrials.gov/). If HDIs can be used to activate canonical Smad signalling in mDA neurons *in vivo*, then they may have neurotrophic effects on mDA neurons and therefore offer a non-invasive therapeutic approach. This will be an important question for future research; however, the difficulty of this approach (as with any small molecule therapy) will be the specific targeting of mDA neurons to limit potential side effects. The studies described above provide proof-of-principle that small molecules can be used to activate BMP–Smad signalling, and thus that other similar molecules may be beneficial in PD.

## Conclusions and future perspectives

As neurotrophic factors can promote mDA neuron survival and axon growth, neurotrophic factor therapy remains promising for PD [17]. Despite the lack of efficacy of intracerebral AAV-NTN in a phase II trial, it is important to note that post hoc analysis found that patients who had been diagnosed within 5 years of receiving AAV-NRTN treatment showed significantly greater improvements in UPDRS scores than those who were diagnosed more than 10 years before [21]. This is an important consideration given that most patients display almost complete mDA axonal loss by 4 years post diagnosis [12]. It suggests that patients at the earlier stages of PD, when some of the nigrostriatal pathway remains, may benefit the most from neurotrophic factor therapy. Allied to this, it will also be important to improve our understanding of the mechanisms of axonal degeneration and neuronal loss in PD. Is axonal degeneration a passive event occurring as a consequence of somal injury, or a distinct process that can be targeted in addition to protecting the soma? If the latter is the case, application of a single neurotrophic factor may not be the best approach. For example, combined overexpression of two neurotrophic factors, cerebral dopamine neurotrophic factor (CDNF) and its paralogue, mesencephalic astrocyte-derived neurotrophic factor (MANF), have synergistic effects on the nigrostriatal pathway in a rat model of PD [85]. However, the receptors and mechanisms of action of these two neurotrophic factors have not yet been well characterized. Given the lack of efficacy of AAV-NTN to date [21] and the fact GDNF ligands may not be able to signal in the PD brain due to down-regulation of Ret [22], it has recently been suggested that ‘better results might be achieved with other trophic factors that are not Ret dependent’ and that ‘it might also be of value to assess trophic factors in animal models that overexpress α-synuclein prior to initiating translational clinical trials’ [21]. Given that BMP ligands have the same efficacy as GDNF in 6-OHDA animal models of PD [18,19], and that their effects on mDA neurons are mediated in a Ret-independent manner through the Smad pathway [34], it will be crucial to determine if BMPR expression or key downstream effector proteins are affected by α-synuclein, and if the BMP ligands can exert neuroprotective effects in the α-synuclein model of PD. It is interesting to note that BMP2 has also recently been shown to up-regulate Nurr1 [86], raising the intriguing possibility that BMP ligands may restore responsiveness to the GDNF ligands, and highlighting the potential for combined neurotrophic factor therapy to protect mDA axons as well as their neuronal soma. In addition, improving our understanding of the molecular mechanisms mediating the neurotrophic effects of BMPs will be an important prerequisite for clinical translation.

## Abbreviations

6-OHDA: 6-hydroxydopamine
AAV: adeno-associated virus
AMH: anti-Mullerian hormone
BBB: blood–brain barrier
BMP: bone morphogenetic protein
BMPR: BMP receptor
BMPR2DN: BMPR2 dominant negative
CTBP: *N*-(4-chloro-3-trifluoromethyl-phenyl)-2-ethoxy-6-pentadecyl-benzamide
GDF: growth and differentiation factor
GDNF: glial cell line-derived neurotrophic factor
HDAC: histone deacetylase
HDI: histone deacetylase inhibitor
mDA: midbrain dopaminergic
MFB: medial forebrain bundle
NTN: neurturin
PD: Parkinson's disease
rh: recombinant human
RRF: retrorubral field
R-Smads: receptor-activated Smad proteins
SNpc: substantia nigra pars compacta
TGFB: transforming growth factor-β
TH: tyrosine hydroxylase
TH^+^: TH-positive
VM: ventral mesencephalon
VTA: ventral tegmental area

## References

[1] Blum M. (1998) A null mutation in TGF-alpha leads to a reduction in midbrain dopaminergic neurons in the substantia nigra. Nat. Neurosci. 1, 374–377 10.1038/1584 10196526

[2] German D.C., Schlusselberg D.S. and Woodward D.J. (1983) Three-dimensional computer reconstruction of midbrain dopaminergic neuronal populations: from mouse to man. J. Neural. Transm. 57, 243–254 10.1007/BF01248996 6140298

[3] Pakkenberg B., Moller A., Gundersen H.J., Mouritzen Dam A. and Pakkenberg H. (1991) The absolute number of nerve cells in substantia nigra in normal subjects and in patients with Parkinson's disease estimated with an unbiased stereological method. J. Neurol. Neurosurg. Psychiatry 54, 30–33 10.1136/jnnp.54.1.30 2010756 PMC1014294

[4] Hegarty S.V., Sullivan A.M. and O'Keeffe G.W. (2013) Midbrain dopaminergic neurons: a review of the molecular circuitry that regulates their development. Dev. Biol. 379, 123–138 10.1016/j.ydbio.2013.04.014 23603197

[5] La Manno G., Gyllborg D., Codeluppi S., Nishimura K., Salto C., Zeisel A. et al. (2016) Molecular diversity of midbrain development in mouse, human, and stem cells. Cell 167, 566–580.e19 10.1016/j.cell.2016.09.027 27716510 PMC5055122

[6] Dahlstroem A. and Fuxe K. (1964) Evidence for the existence of monoamine-containing neurons in the central nervous system. I. Demonstration of monoamines in the cell bodies of brain stem neurons. Acta Physiol. Scand. Suppl. 232, 1–55 14229500

[7] Tzschentke T.M. and Schmidt W.J. (2000) Functional relationship among medial prefrontal cortex, nucleus accumbens, and ventral tegmental area in locomotion and reward. Crit. Rev. Neurobiol. 14, 131–142 10.1615/CritRevNeurobiol.v14.i2.20 11513242

[8] Meyer-Lindenberg A., Miletich R.S., Kohn P.D., Esposito G., Carson R.E., Quarantelli M. et al. (2002) Reduced prefrontal activity predicts exaggerated striatal dopaminergic function in schizophrenia. Nat. Neurosci. 5, 267–271 10.1038/nn804 11865311

[9] Robinson T.E. and Berridge K.C. (1993) The neural basis of drug craving: an incentive-sensitization theory of addiction. Brain Res. Brain Res. Rev. 18, 247–291 10.1016/0165-0173(93)90013-P 8401595

[10] Clemens K.J., Castino M.R., Cornish J.L., Goodchild A.K. and Holmes N.M. (2014) Behavioral and neural substrates of habit formation in rats intravenously self-administering nicotine. Neuropsychopharmacology 39, 2584–2593 10.1038/npp.2014.111 24823947 PMC4207338

[11] Wise R.A. (2009) Roles for nigrostriatal–not just mesocorticolimbic–dopamine in reward and addiction. Trends Neurosci. 32, 517–524 10.1016/j.tins.2009.06.004 19758714 PMC2755633

[12] Kordower J.H., Olanow C.W., Dodiya H.B., Chu Y., Beach T.G., Adler C.H. et al. (2013) Disease duration and the integrity of the nigrostriatal system in Parkinson's disease. Brain 136, (Pt 8)2419–2431 10.1093/brain/awt192 23884810 PMC3722357

[13] Hirsch E., Graybiel A.M. and Agid Y.A. (1988) Melanized dopaminergic neurons are differentially susceptible to degeneration in Parkinson's disease. Nature 334, 345–348 10.1038/334345a0 2899295

[14] Damier P., Hirsch E.C., Agid Y. and Graybiel A.M. (1999) The substantia nigra of the human brain. II. Patterns of loss of dopamine-containing neurons in Parkinson's disease. Brain 122, (Pt 8)1437–1448 10.1093/brain/122.8.1437 10430830

[15] Parkinson J. (1817) An essay on the shaking palsy. J. Neuropsychiatry Clin. Neurosci. 14, 223–236 10.1176/jnp.14.2.223 11983801

[16] Drew L. (2016) Two hundred steps. Nature 538, S2–S3 10.1038/538S2a 27783577

[17] Sullivan A.M. and O'Keeffe G.W. (2016) Neurotrophic factor therapy for Parkinson's disease: past, present and future. Neural. Regen. Res. 11, 205–207 10.4103/1673-5374.177710 27073356 PMC4810967

[18] Hegarty S.V., Sullivan A.M. and O'Keeffe G.W. (2014) Roles for the TGFbeta superfamily in the development and survival of midbrain dopaminergic neurons. Mol. Neurobiol. 50, 559–573 10.1007/s12035-014-8639-3 24504901

[19] Kelly M.J., O'Keeffe G.W. and Sullivan A.M. (2015) Viral vector delivery of neurotrophic factors for Parkinson's disease therapy. Expert Rev. Mol. Med. 17, e8 10.1017/erm.2015.6 25997719

[20] Hegarty S.V., Lee D., O'Keeffe G.W. and Sullivan A.M. (2017) Effects of intracerebral neurotrophic factor application on motor symptoms in Parkinson's disease: a systematic review and meta-analysis. Parkinsonism Relat. Disord. in press 10.1016/j.parkreldis.2017.02.011 28215730

[21] Warren Olanow C., Bartus R.T., Baumann T.L., Factor S., Boulis N., Stacy M. et al. (2015) Gene delivery of neurturin to putamen and substantia nigra in Parkinson disease: a double-blind, randomized, controlled trial. Ann. Neurol. 78, 248–257 10.1002/ana.24436 26061140

[22] Decressac M., Kadkhodaei B., Mattsson B., Laguna A., Perlmann T. and Bjorklund A. (2012) alpha-Synuclein-induced down-regulation of Nurr1 disrupts GDNF signaling in nigral dopamine neurons. Sci. Transl. Med. 4, 163ra56 10.1126/scitranslmed.3004676 23220632

[23] Decressac M., Ulusoy A., Mattsson B., Georgievska B., Romero-Ramos M., Kirik D. et al. (2011) GDNF fails to exert neuroprotection in a rat alpha-synuclein model of Parkinson's disease. Brain 134, Pt 8 2302–2311 10.1093/brain/awr149 21712347

[24] Weiss A. and Attisano L. (2013) The TGFbeta superfamily signaling pathway. Wiley Interdiscip. Rev. Dev. Biol. 2, 47–63 10.1002/wdev.86 23799630

[25] Hegarty S.V., O'Keeffe G.W. and Sullivan A.M. (2013) BMP-Smad 1/5/8 signalling in the development of the nervous system. Prog. Neurobiol. 109, 28–41 10.1016/j.pneurobio.2013.07.002 23891815

[26] Roussa E., von Bohlen und Halbach O. and Krieglstein K. (2009) TGF-beta in dopamine neuron development, maintenance and neuroprotection. Adv. Exp. Med. Biol. 651, 81–90 10.1007/978-1-4419-0322-8 19731553

[27] Yin H., Yeh L.C., Hinck A.P. and Lee J.C. (2008) Characterization of ligand-binding properties of the human BMP type II receptor extracellular domain. J. Mol. Biol. 378, 191–203 10.1016/j.jmb.2008.02.031 18342887

[28] Weber D., Kotzsch A., Nickel J., Harth S., Seher A., Mueller U. et al. (2007) A silent H-bond can be mutationally activated for high-affinity interaction of BMP-2 and activin type IIB receptor. BMC Struct. Biol. 7, 6 10.1186/1472-6807-7-6 17295905 PMC1802081

[29] Koenig B.B., Cook J.S., Wolsing D.H., Ting J., Tiesman J.P., Correa P.E. et al. (1994) Characterization and cloning of a receptor for BMP-2 and BMP-4 from NIH 3T3 cells. Mol. Cell Biol. 14, 5961–5974 10.1128/MCB.14.9.5961 8065329 PMC359122

[30] Nishitoh H., Ichijo H., Kimura M., Matsumoto T., Makishima F., Yamaguchi A. et al. (1996) Identification of type I and type II serine/threonine kinase receptors for growth/differentiation factor-5. J. Biol. Chem. 271, 21345–21352 10.1074/jbc.271.35.21345 8702914

[31] Lee-Hoeflich S.T., Causing C.G., Podkowa M., Zhao X., Wrana J.L. and Attisano L. (2004) Activation of LIMK1 by binding to the BMP receptor, BMPRII, regulates BMP-dependent dendritogenesis. EMBO J. 23, 4792–4801 10.1038/sj.emboj.7600418 15538389 PMC535083

[33] Guan J., Li H., Lv T., Chen D., Yuan Y. and Qu S. (2014) Bone morphogenic protein-7 contributes to cerebral ischemic preconditioning induced-ischemic tolerance by activating p38 mitogen-activated protein kinase signaling pathway. Inflammation 37, 1289–1296 10.1007/s10753-014-9856-7 24682853

[34] Hegarty S.V., Collins L.M., Gavin A.M., Roche S.L., Wyatt S.L., Sullivan A.M. et al. (2014) Canonical BMP-Smad signalling promotes neurite growth in rat midbrain dopaminergic neurons. Neuromol. Med. 16, 473–489 10.1007/s12017-014-8299-5 24682653

[35] Hegarty S.V., Sullivan A.M. and O'Keeffe G.W. (2013) BMP2 and GDF5 induce neuronal differentiation through a Smad dependant pathway in a model of human midbrain dopaminergic neurons. Mol. Cell Neurosci. 56, 263–271 10.1016/j.mcn.2013.06.006 23831389

[36] Chou J., Harvey B.K., Ebendal T., Hoffer B. and Wang Y. (2008) Nigrostriatal alterations in bone morphogenetic protein receptor II dominant negative mice. Acta Neurochir. Suppl. 101, 93–98 10.1007/978-3-211-78205-7 18642641 PMC2572854

[37] Schneider C., Wicht H., Enderich J., Wegner M. and Rohrer H. (1999) Bone morphogenetic proteins are required in vivo for the generation of sympathetic neurons. Neuron 24, 861–870 10.1016/S0896-6273(00)81033-8 10624949

[38] O'Keeffe G.W., Gutierrez H., Howard L., Laurie C.W., Osorio C., Gavalda N. et al. (2016) Region-specific role of growth differentiation factor-5 in the establishment of sympathetic innervation. Neural. Dev. 11, 4 10.1186/s13064-016-0060-3 26878848 PMC4755026

[39] Storm E.E., Huynh T.V., Copeland N.G., Jenkins N.A., Kingsley D.M. and Lee S.J. (1994) Limb alterations in brachypodism mice due to mutations in a new member of the TGF beta-superfamily. Nature 368, 639–643 10.1038/368639a0 8145850

[40] Kopra J., Vilenius C., Grealish S., Harma M.A., Varendi K., Lindholm J. et al. (2015) GDNF is not required for catecholaminergic neuron survival in vivo. Nat. Neurosci. 18, 319–322 10.1038/nn.3941 25710828 PMC4878654

[41] Chou J., Luo Y., Kuo C.C., Powers K., Shen H., Harvey B.K. et al. (2008) Bone morphogenetic protein-7 reduces toxicity induced by high doses of methamphetamine in rodents. Neuroscience 151, 92–103 10.1016/j.neuroscience.2007.10.044 18082966 PMC6167133

[42] Tagliaferro P. and Burke R.E. (2016) Retrograde axonal degeneration in Parkinson disease. J. Parkinson Dis. 6, 1–15 10.3233/JPD-150769 PMC4927911 27003783

[43] Cheng H.C., Ulane C.M. and Burke R.E. (2010) Clinical progression in Parkinson disease and the neurobiology of axons. Ann. Neurol. 67, 715–725 10.1002/ana.21995 20517933 PMC2918373

[44] Matsuda W., Furuta T., Nakamura K.C., Hioki H., Fujiyama F., Arai R. et al. (2009) Single nigrostriatal dopaminergic neurons form widely spread and highly dense axonal arborizations in the neostriatum. J. Neurosci. 29, 444–453 10.1523/JNEUROSCI.4029-08.2009 19144844 PMC6664950

[45] Sauer H. and Oertel W.H. (1994) Progressive degeneration of nigrostriatal dopamine neurons following intrastriatal terminal lesions with 6-hydroxydopamine: a combined retrograde tracing and immunocytochemical study in the rat. Neuroscience 59, 401–415 10.1016/0306-4522(94)90605-X 7516500

[46] Ries V., Silva R.M., Oo T.F., Cheng H.C., Rzhetskaya M., Kholodilov N. et al. (2008) JNK2 and JNK3 combined are essential for apoptosis in dopamine neurons of the substantia nigra, but are not required for axon degeneration. J. Neurochem. 107, 1578–1588 10.1111/j.1471-4159.2008.05713.x 19014392 PMC2632584

[47] Tagliaferro P., Kareva T., Oo T.F., Yarygina O., Kholodilov N. and Burke R.E. (2015) An early axonopathy in a hLRRK2(R1441G) transgenic model of Parkinson disease. Neurobiol. Dis. 82, 359–371 10.1016/j.nbd.2015.07.009 26192625 PMC4640977

[48] Burke R.E. and O'Malley K. (2013) Axon degeneration in Parkinson's disease. Exp. Neurol. 246, 72–83 10.1016/j.expneurol.2012.01.011 22285449 PMC3340476

[49] Jordan J., Bottner M., Schluesener H.J., Unsicker K. and Krieglstein K. (1997) Bone morphogenetic proteins: neurotrophic roles for midbrain dopaminergic neurons and implications of astroglial cells. Eur. J. Neurosci. 9, 1699–1709 10.1111/j.1460-9568.1997.tb01527.x 9283824

[50] Reiriz J., Espejo M., Ventura F., Ambrosio S. and Alberch J. (1999) Bone morphogenetic protein-2 promotes dissociated effects on the number and differentiation of cultured ventral mesencephalic dopaminergic neurons. J. Neurobiol. 38, 161–170 10.1002/(SICI)1097-4695(19990205)38:2<161::AID-NEU1>3.0.CO;2-3 10022564

[51] Krieglstein K., Suter-Crazzolara C., Hötten G., Pohl J. and Unsicker K. (1995) Trophic and protective effects of growth/differentiation factor 5, a member of the transforming growth factor-beta superfamily, on midbrain dopaminergic neurons. J. Neurosci. Res. 42, 724–732 10.1002/jnr.490420516 8600306

[52] O'Keeffe G.W., Dockery P. and Sullivan A.M. (2004) Effects of growth/differentiation factor 5 on the survival and morphology of embryonic rat midbrain dopaminergic neurones in vitro. J. Neurocytol. 33, 479–488 10.1007/s11068-004-0511-y 15906156

[53] O'Sullivan D.B., Harrison P.T. and Sullivan A.M. (2010) Effects of GDF5 overexpression on embryonic rat dopaminergic neurones in vitro and in vivo. J. Neural. Transm. 117, 559–572 10.1007/s00702-010-0392-9 20349094

[54] Wood T.K., McDermott K.W. and Sullivan A.M. (2005) Differential effects of growth/differentiation factor 5 and glial cell line-derived neurotrophic factor on dopaminergic neurons and astroglia in cultures of embryonic rat midbrain. J. Neurosci. Res. 80, 759–766 10.1002/jnr.20507 15880784

[55] Jaumotte J.D. and Zigmond M.J. (2014) Comparison of GDF5 and GDNF as neuroprotective factors for postnatal dopamine neurons in ventral mesencephalic cultures. J. Neurosci. Res. 92, 1425–1433 10.1002/jnr.23425 24916473

[56] Xie H.R., Hu L.S. and Li G.Y. (2010) SH-SY5Y human neuroblastoma cell line: in vitro cell model of dopaminergic neurons in Parkinson's disease. Chin. Med. J. (Engl.) 123, 1086–1092 20497720

[57] Hegarty S.V., Sullivan A.M. and O'Keeffe G.W. (2016) Protocol for evaluation of neurotrophic strategies in Parkinson's disease related dopaminergic and sympathetic neurons in vitro. J. Biol. Methods 3, e50 DOI: 10.14440/jbm.2016.124 31453215 PMC6706149

[58] Toulouse A., Collins G.C. and Sullivan A.M. (2012) Neurotrophic effects of growth/differentiation factor 5 in a neuronal cell line. Neurotox. Res. 21, 256–265 10.1007/s12640-011-9266-7 21858606

[59] Yu P.B., Hong C.C., Sachidanandan C., Babitt J.L., Deng D.Y., Hoyng S.A. et al. (2008) Dorsomorphin inhibits BMP signals required for embryogenesis and iron metabolism. Nat. Chem. Biol. 4, 33–41 10.1038/nchembio.2007.54 18026094 PMC2727650

[60] Fujii M., Takeda K., Imamura T., Aoki H., Sampath T.K., Enomoto S. et al. (1999) Roles of bone morphogenetic protein type I receptors and Smad proteins in osteoblast and chondroblast differentiation. Mol. Biol. Cell 10, 3801–3813 10.1091/mbc.10.11.3801 10564272 PMC25680

[61] Sullivan A.M., Opacka-Juffry J., Hotten G., Pohl J. and Blunt S.B. (1997) Growth/differentiation factor 5 protects nigrostriatal dopaminergic neurones in a rat model of Parkinson's disease. Neurosci. Lett. 233, 73–76 10.1016/S0304-3940(97)00623-X 9350835

[62] Sullivan A.M., Opacka-Juffry J., Pohl J. and Blunt S.B. (1999) Neuroprotective effects of growth/differentiation factor 5 depend on the site of administration. Brain Res. 818, 176–179 10.1016/S0006-8993(98)01275-X 9914454

[63] Hurley F.M., Costello D.J. and Sullivan A.M. (2004) Neuroprotective effects of delayed administration of growth/differentiation factor-5 in the partial lesion model of Parkinson's disease. Exp. Neurol. 185, 281–289 10.1016/j.expneurol.2003.10.003 14736509

[64] Costello D.J., O'Keeffe G.W., Hurley F.M. and Sullivan A.M. (2012) Transplantation of novel human GDF5-expressing CHO cells is neuroprotective in models of Parkinson's disease. J. Cell Mol. Med. 16, 2451–2460 10.1111/j.1582-4934.2012.01562.x 22436046 PMC3823439

[65] Zuch C.L., David D., Ujhelyi L., Hudson J.L., Gerhardt G.A., Kaplan P.L. et al. (2004) Beneficial effects of intraventricularly administered BMP-7 following a striatal 6-hydroxydopamine lesion. Brain Res. 1010, 10–16 10.1016/j.brainres.2003.12.058 15126112

[66] Harvey B.K., Mark A., Chou J., Chen G.J., Hoffer B.J. and Wang Y. (2004) Neurotrophic effects of bone morphogenetic protein-7 in a rat model of Parkinson's disease. Brain Res. 1022, 88–95 10.1016/j.brainres.2004.06.072 15353217

[67] Sullivan A.M., Pohl J. and Blunt S.B. (1998) Growth/differentiation factor 5 and glial cell line-derived neurotrophic factor enhance survival and function of dopaminergic grafts in a rat model of Parkinson's disease. Eur. J. Neurosci. 10, 3681–3688 10.1046/j.1460-9568.1998.00378.x 9875347

[68] Espejo M., Cutillas B., Ventura F. and Ambrosio S. (1999) Exposure of foetal mesencephalic cells to bone morphogenetic protein-2 enhances the survival of dopaminergic neurones in rat striatal grafts. Neurosci. Lett. 275, 13–16 10.1016/S0304-3940(99)00708-9 10554973

[69] Hegarty S.V., O'Keeffe G.W. and Sullivan A.M. (2014) Neurotrophic factors: from neurodevelopmental regulators to novel therapies for Parkinson's disease. Neural. Regen. Res. 9, 1708–1711 10.4103/1673-5374.143410 25422631 PMC4238158

[70] Bartus R.T., Baumann T.L., Siffert J., Herzog C.D., Alterman R., Boulis N. et al. (2013) Safety/feasibility of targeting the substantia nigra with AAV2-neurturin in Parkinson patients. Neurology 80, 1698–1701 10.1212/WNL.0b013e3182904faa 23576625 PMC3716474

[71] Bartus R.T., Brown L., Wilson A., Kruegel B., Siffert J., Johnson E.M. Jr et al. (2011) Properly scaled and targeted AAV2-NRTN (neurturin) to the substantia nigra is safe, effective and causes no weight loss: support for nigral targeting in Parkinson's disease. Neurobiol. Dis. 44, 38–52 10.1016/j.nbd.2011.05.026 21704161

[72] Chan H.M. and La Thangue N.B. (2001) p300/CBP proteins: HATs for transcriptional bridges and scaffolds. J. Cell Sci. 114, Pt 13 2363–2373 11559745 10.1242/jcs.114.13.2363

[73] Pouponnot C., Jayaraman L. and Massague J. (1998) Physical and functional interaction of SMADs and p300/CBP. J. Biol. Chem. 273, 22865–22868 10.1074/jbc.273.36.22865 9722503

[74] Pearson K.L., Hunter T. and Janknecht R. (1999) Activation of Smad1-mediated transcription by p300/CBP. Biochim. Biophys. Acta 1489, 354–364 10.1016/S0167-4781(99)00166-9 10673036

[75] Balasubramanyam K., Swaminathan V., Ranganathan A. and Kundu T.K. (2003) Small molecule modulators of histone acetyltransferase p300. J. Biol. Chem. 278, 19134–19140 10.1074/jbc.M301580200 12624111

[76] Hegarty S.V., O'Leary E., Solger F., Stanicka J., Sullivan A.M. and O'Keeffe G.W. (2016) A small molecule activator of p300/CBP histone acetyltransferase promotes survival and neurite growth in a cellular model of Parkinson's disease. Neurotox. Res. 30, 510–520 10.1007/s12640-016-9636-2 27256286

[77] Selvi B.R., Jagadeesan D., Suma B.S., Nagashankar G., Arif M., Balasubramanyam K. et al. (2008) Intrinsically fluorescent carbon nanospheres as a nuclear targeting vector: delivery of membrane-impermeable molecule to modulate gene expression in vivo. Nano Lett. 8, 3182–3188 10.1021/nl801503m 18800851

[78] Seo Y.J., Kang Y., Muench L., Reid A., Caesar S., Jean L. et al. (2014) Image-guided synthesis reveals potent blood-brain barrier permeable histone deacetylase inhibitors. ACS Chem. Neurosci. 5, 588–596 10.1021/cn500021p 24780082 PMC4102966

[79] Gardian G., Yang L., Cleren C., Calingasan N.Y., Klivenyi P. and Beal M.F. (2004) Neuroprotective effects of phenylbutyrate against MPTP neurotoxicity. Neuromol. Med. 5, 235–241 10.1385/NMM:5:3:235 15626823

[80] Kidd S. and Schneider J. (2011) Protective effects of valproic acid on the nigrostriatal dopamine system in a 1-methyl-4-phenyl-1, 2, 3, 6-tetrahydropyridine mouse model of Parkinson's disease. Neuroscience 194, 189–194 10.1016/j.neuroscience.2011.08.010 21846494 PMC3196607

[81] Laurent R.S., O'Brien L. and Ahmad S. (2013) Sodium butyrate improves locomotor impairment and early mortality in a rotenone-induced Drosophila model of Parkinson's disease. Neuroscience 246, Complete 382–390 10.1016/j.neuroscience.2013.04.037 23623990 PMC3721507

[82] Kontopoulos E., Parvin J.D. and Feany M.B. (2006) α-synuclein acts in the nucleus to inhibit histone acetylation and promote neurotoxicity. Hum. Mol. Genet. 15, 3012–3023 10.1093/hmg/ddl243 16959795

[83] Outeiro T.F., Kontopoulos E., Altmann S.M., Kufareva I., Strathearn K.E., Amore A.M. et al. (2007) Sirtuin 2 inhibitors rescue α-synuclein-mediated toxicity in models of Parkinson's disease. Science 317, 516–519 10.1126/science.1143780 17588900

[84] Collins L.M., Adriaanse L.J., Theratile S.D., Hegarty S.V., Sullivan A.M. and O'Keeffe G.W. (2015) Class-IIa histone deacetylase inhibition promotes the growth of neural processes and protects them against neurotoxic insult. Mol. Neurobiol. 51, 1432–1442 10.1007/s12035-014-8820-8 25065734

[85] Cordero-Llana O., Houghton B.C., Rinaldi F., Taylor H., Yanez-Munoz R.J., Uney J.B. et al. (2015) Enhanced efficacy of the CDNF/MANF family by combined intranigral overexpression in the 6-OHDA rat model of Parkinson's disease. Mol. Ther. 23, 244–254 10.1038/mt.2014.206 25369767 PMC4445614

[86] Yan W., Chen Z.Y., Chen J.Q. and Chen H.M. (2016) BMP2 promotes the differentiation of neural stem cells into dopaminergic neurons in vitro via miR-145-mediated upregulation of Nurr1 expression. Am. J. Transl. Res. 8, 3689–3699 27725851 PMC5040669

[87] Uhlen M., Fagerberg L., Hallstrom B.M., Lindskog C., Oksvold P., Mardinoglu A. et al. (2015) Proteomics. Tissue-based map of the human proteome. Science 347, 1260419 10.1126/science.1260419 25613900

